# Matrix-assisted Laser Desorption Ionization-Time of Flight Mass Spectrometry (MALDI-TOF MS) Can Precisely Discriminate the Lineages of *Listeria monocytogenes* and Species of *Listeria*

**DOI:** 10.1371/journal.pone.0159730

**Published:** 2016-07-21

**Authors:** Teruyo Ojima-Kato, Naomi Yamamoto, Hajime Takahashi, Hiroto Tamura

**Affiliations:** 1 Knowledge Hub Aichi, Aichi Science and Technology Foundation, Yakusa, Toyota, Aichi 470-0356, Japan; 2 School of Agriculture, Meijo University, Shiogamaguchi, Tenpaku-ku, Nagoya, Aichi 468-0073, Japan; 3 Department of Food Science and Technology, Faculty of Marine Science, Tokyo University of Marine Science and Technology, 4-5-7, Konan, Minato-ku, Tokyo, 108-8477 Japan; INRA Clermont-Ferrand Research Center, FRANCE

## Abstract

The genetic lineages of *Listeria monocytogenes* and other species of the genus *Listeria* are correlated with pathogenesis in humans. Although matrix-assisted laser desorption ionization-time of flight mass spectrometry (MALDI-TOF MS) has become a prevailing tool for rapid and reliable microbial identification, the precise discrimination of *Listeria* species and lineages remains a crucial issue in clinical settings and for food safety. In this study, we constructed an accurate and reliable MS database to discriminate the lineages of *L*. *monocytogenes* and the species of *Listeria* (*L*. *monocytogenes*, *L*. *innocua*, *L*. *welshimeri*, *L*. *seeligeri*, *L*. *ivanovii*, *L*. *grayi*, and *L*. *rocourtiae*) based on the *S10-spc-alpha* operon gene encoded ribosomal protein mass spectrum (*S10*-GERMS) proteotyping method, which relies on both genetic information (genomics) and observed MS peaks in MALDI-TOF MS (proteomics). The specific set of eight biomarkers (ribosomal proteins L24, L6, L18, L15, S11, S9, L31 type B, and S16) yielded characteristic MS patterns for the lineages of *L*. *monocytogenes* and the different species of *Listeria*, and led to the construction of a MS database that was successful in discriminating between these organisms in MALDI-TOF MS fingerprinting analysis followed by advanced proteotyping software Strain Solution analysis. We also confirmed the constructed database on the proteotyping software Strain Solution by using 23 *Listeria* strains collected from natural sources.

## Introduction

The genus *Listeria* is gram-positive bacterium that can grow in saline and cold environments [1]. At present, the bacterial genus *Listeria* consists of 17 species, including *Listeria monocytogenes*, *L*. *innocua*, *L*. *welshimeri*, *L*. *seeligeri*, *L*. *ivanovii*, and *L*. *grayi*, which have been recognized since the 1960s to 1980s, and other recently emerging species, namely *L*. *marthii*, *L*. *recourtiae*, *L*. *floridensis*, *L*. *aquatica*, *L*. *fleischmannii*, *L*. *newyorkensis*, *L*. *cornellensis*, *L*. *weihenstephanensis*, *L*. *grandensis*, *L*. *riparia*, *and L*. *booriae* (http://www.bacterio.net/) [2, 3]. Among these, *L*. *monocytogenes* and *L*. *ivanovii* are known to be pathogenic in warm-blooded hosts. Recently, *L*. *innocua* was reported to have pathogenic potential [4]. Most cases of human diseases are associated with *L*. *monocytogenes*, which causes severe listeriosis through infections from ready-to-eat foods [5]. *L*. *monocytogenes* can be further divided into four evolutionary lineages of 13 serotypes: lineage I (serotypes 1/2b, 3b, 4b, 4d, and 4e), lineage II (serotypes 1/2a, 1/2c, 3a, and 3c), and lineages III (serotypes 4a and 4c) and IV (4a, 4b, 4c) [6, 7]. Serotypes 4b, 1/2a, and 1/2b, which belong to lineages I and II, are commonly isolated from human patients [8], while lineages III and IV from the ruminants [6, 9].

Detection of *Listeria* species and serotypes of *L*. *monocytogenes* is crucial for clinical and food safety. Traditionally, culture-based methods using chromogenic media have been utilized in food laboratories to differentiate *L*. *monocytogenes* from other *Listeria* species [10]. Serotyping of *L*. *monocytogenes* is carried out by immunological methods using antisera [11]. Alternative advanced typing methods, such as pulsed-field gel electrophoresis [12], multilocus sequence typing [13, 14], and DNA microarray [15], have been reported. Although reliable typing can be performed using these methods, they are laborious and time-consuming.

Matrix-assisted laser desorption ionization-time of flight mass spectrometry (MALDI-TOF MS) has become a standard tool for rapid and reliable microbial identification in clinical and food laboratories. Typing trials of *Listeria* species and *L*. *monocytogenes* have been reported by several research groups so far [16–19]. Barbuddhe *et al*. reported that *L*. *monocytogenes*, *L*. *innocua*, *L*. *welshimeri*, *L*. *ivanovii* and *L*. *seeligeri* could be discriminated by a MALDI TOF-MS fingerprinting method that compares obtained profile spectra and registered reference spectral database to calculate a matching score. They also identified that detection of MS peak at mass to ion ratios (*m*/*z*) of 5590 and 11179 could differentiate between serotype 4a and 4c of *L*. *monocytogenes* [16]. In contrast, a study by Farfour *et al*. revealed that identification of *Listeria* spp. could only be achieved at the genus level, except for *L*. *grayi* using the MALDI TOF MS Andromas system [17]. Hsueh *et al*. reported the detected five MS peaks at *m/z* 5594.85, 6184.39, 11871.31, 5601.21, and 11199.33 could be used to distinguish serotypes 1/2a (lineage II), and 1/2b and 4b (lineage I) of *L*. *monocytogenes* [19]. These results from several groups demonstrate that the MS sizes of the detected peaks differ between research groups, and that the proteins were not fully identified, which potentially leads to data inconsistency.

The *S10-spc-alpha* operon gene encoded ribosomal protein mass spectrum (*S10*-GERMS) method provides an accurate and reliable *m*/*z* database of biomarker proteins encoded in the *S10-spc-alpha* operon and characteristic genes. The *m*/*z* values in the database are confirmed by genomic information and experimentally observed MS peaks in MALDI TOF-MS analysis [20]. The *m*/*z* database in this method reflects the subtle *m*/*z* differences that occur with even a single amino acid substitution within the selected biomarker proteins of closely related species. Therefore, the *S10*-GERMS method can realize microbial identification at a higher resolution than the conventional fingerprinting approaches, and has been successfully applied to species, pathovar, or serotype level discrimination of various industrially important bacteria, such as *Bacillus* spp., *Pseudomonas putida*, *P*. *syringae*, *Lactobacillus casei*, and the enterohemorrhagic *Escherichia coli* [21–24]. To realize a fully automatic analysis based on the *S10*-GERMS method, our group has developed the novel external software ‘Strain Solution’ for MALDI-TOF MS analysis [25], and has made it available from the Shimadzu Corporation (Kyoto, Japan).

Here, we report the construction of an accurate and reliable *m*/*z* database of annotated biomarker proteins for *Listeria* spp. based on the *S10*-GERMS method. We demonstrate that the selected biomarker proteins from the database can fulfill our aim in discriminating lineages of *Listeria monocytogenes*, and species of the genus *Listeria* (*L*. *monocytogenes*, *L*. *innocua*, *L*. *welshimeri*, *L*. *seeligeri*, *L*. *ivanovii*, *L*. *grayi*, and *L*. *rocourtiae*) using MALDI-TOF MS analysis.

## Materials and Methods

### Bacterial strains

For constructing the theoretical MS database, we used publically available *L*. *monocytogenes* strains and *Listeria* spp. listed in Table 1 obtained from the Japan Collection of Microorganisms, RIKEN BRC (Tsukuba, Japan), the American Type Culture Collection (Manassas, VA, USA), and the National BioResource Project GTC Collection (Gifu, Japan). They were aerobically cultivated in the brain heart infusion medium (Becton Dickinson, Franklin Lakes, NJ, USA) at 30°C. *L*. *monocytogenes* strains were serotyped using the Agglutinating Sera *Listeria* Antisera Set (Denka Seiken, Tokyo, Japan) and multiplex PCR as a countercheck [26].

**Table 1:** Bacterial strains used to construct an MS database.

No.	Genus	Species	Subspecies	Strain	Serotype^a^	Lineage	Source^b^
1	*Listeria*	*monocytogenes*		ATCC15313^T^	1/2a^c^	II	ATCC
2	*Listeria*	*monocytogenes*		JCM2873	4d	I	JCM
3	*Listeria*	*monocytogenes*		JCM7671	1/2a	II	JCM
4	*Listeria*	*monocytogenes*		JCM7672	1/2c	II	JCM
5	*Listeria*	*monocytogenes*		JCM7673	3a	II	JCM
6	*Listeria*	*monocytogenes*		JCM7674	4a	III	JCM
7	*Listeria*	*monocytogenes*		JCM7675	4b	I	JCM
8	*Listeria*	*monocytogenes*		JCM7676	1/2b	I	JCM
9	*Listeria*	*monocytogenes*		JCM7677	3b	I	JCM
10	*Listeria*	*monocytogenes*		JCM7678	3c	II	JCM
11^e^	*Listeria*	*seeligeri*		JCM7679			JCM
12	*Listeria*	*monocytogenes*		JCM7680	4d	I	JCM
13^e^	*Listeria*	*seeligeri*		JCM7682			JCM
14	*Listeria*	*monocytogenes*		JCM7683	3b	I	JCM
15	*Listeria*	*monocytogenes*		ATCC51772	1/2a	II	ATCC
16	*Listeria*	*monocytogenes*		ATCC19115	4b	I	ATCC
17	*Listeria*	*innocua*		ATCC33090^T^	6a^d^		ATCC
18	*Listeria*	*innocua*		GTC02960			NBRP
19	*Listeria*	*ivanovii*	*ivanovii*	JCM7681			JCM
20	*Listeria*	*ivanovii*	*londoniensis*	ATCC49954			ATCC
21	*Listeria*	*seeligeli*		ATCC35967^T^			ATCC
22	*Listeria*	*welshimeri*		GTC02963	6b^d^		NBRP
23	*Listeria*	*grayi*		ATCC19120^T^			ATCC
24	*Listeria*	*rocourtiae*		GTC16429^T^			NBRP

^a^Serotypes of *L*. *monocytogenes* were determined using an agglutination test with antisera and PCR.

^b^ATCC: American Type Culture Collection, JCM: Japan Collection of Microorganisms, RIKEN BRC, NBRP: National BioResource Project.

^c^weak agglutination reaction with H antisera from Denka Seiken.

^d^Serotype information for strains No. 17 and 22 is provided by the supplier.

^e^These strains (JCM7689 and JCM7682) were deposited as *L*. *monocytogenes* in JCM but we revealed that they are *L*. *seeligeri*.

To evaluate the constructed MS database, environmental isolates of *Listeria* species (*L*. *monocytogenes*, *L*. *innocua*, *L*. *ivanovii*, *L*. *seeligeri*, and *L*. *rocourtiae*) listed in Table 2 were used. They were screened from river water in Japan and identified by the conventional method using ALOA media (bioMérieux, Lyon, France) and antisera serotyping kit. Species of *Listeria* were determined by a physiological biochemical test using the *Listeria* identification system Api (bioMérieux), and 16S rRNA sequencing [27]. Pathogenicity of *L*. *monocytogenes* was confirmed by the CAMP test [28] and multiplex PCR for the *inlB*, *plcA*, *plcB*, and *clpE* genes [29]. Serotypes of *L*. *innocua* were not defined because antisera for serotyping were not available.

**Table 2:** Bacterial strains used to evaluate the constructed MS database.

No	Genus	Species	ID code	Serotype (lineage)	Source
1	*Listeria*	*monocytogenes*	2009.6.15–2	4b (I)	River water
2	*Listeria*	*monocytogenes*	2009.6.9–1	4b (I)	River water
3	*Listeria*	*monocytogenes*	2009.6.1–2	4b (I)	River water
4	*Listeria*	*monocytogenes*	12.9.11.1–1	4b (I)	River water
5	*Listeria*	*monocytogenes*	12.9.11.3–1	1/2a (I)	River water
6	*Listeria*	*monocytogenes*	12.10.15.1–1	1/2b (I)	River water
7	*Listeria*	*innocua*	12.9.11.4–4		River water
8	*Listeria*	*innocua*	12.10.15.5–3		River water
9	*Listeria*	*innocua*	12.10.15.2–3		River water
10	*Listeria*	*innocua*	2009.6.9–2		River water
11	*Listeria*	*innocua*	2009.6.5–3		River water
12	*Listeria*	*innocua*	2009.6.3–4		River water
13	*Listeria*	*seeligeri*	2009.6.4–2		River water
14	*Listeria*	*seeligeri*	2009.6.5–4		River water
15	*Listeria*	*seeligeri*	12.9.11.5–3		River water
16	*Listeria*	*seeligeri*	12.9.11.4–3		River water
17	*Listeria*	*monocytogenes*	12.9.11.2–3	4b (I)	River water
18	*Listeria*	*rocourtiae*	12.10.15.4–3		River water
19	*Listeria*	*rocourtiae*	12.9.26.10–3		River water
20	*Listeria*	*rocourtiae*	12.9.26.6–3		River water
21	*Listeria*	*ivanovii*	2009.6.12–3		River water
22	*Listeria*	*ivanovii*	2009.6.8–2		River water
23	*Listeria*	*ivanovii*	2009.6.2		River water

### Construction of an MS database

The primers for DNA sequence analysis were designed based on the consensus sequence information available from the Kyoto Encyclopedia of Genes and Genomes (KEGG, http://www.genome.jp/kegg/) and NCBI (http://www.ncbi.nlm.nih.gov/) (Table 3). DNA fragments for sequence analysis were amplified by PCR using KOD plus DNA polymerase (Toyobo, Osaka, Japan) and extracted genomic DNA as the template as described previously [24]. We used the Big Dye ver. 3.1 Cycle Sequencing Kit (Applied Biosystems, Foster City, CA, USA) for sequencing, and calculated the theoretical *m*/*z* values of biomarker proteins using an in-house macro software programmed based on the workflow of DNA to amino acid conversion, calculating protein MS from each amino acid, and addition of a proton (*m*/*z* 1.01) and reducing MS of N-terminal Met (*m*/*z* 131.19) when the second amino acid is Ala, Cys, Gly, Pro, Ser, Thr, or Val.

**Table 3:** DNA Primers used in this study.

Name	Sequence (5'-3')	Purpose
Lm-S10-1	CATGGCGGATGTTCAGGTAA	Amplification of *S10* region and sequencing
Lm-S10-R	CTCCTTCCAGAATAACGGGT	Amplification of *S10* region and sequencing
Lm-S10-2	AGCAGCACAAAACGTGGTAC	Sequencing of *S10* region
Lm-S10-3	AAGGAGGACTAACGAATGCC	Sequencing of *S10* region
Lm-S10-4	TGCACGCAACTTACAAGGCA	Sequencing of *S10* region
Lm-S10-5	CGGACGCAATAACCAAGGTA	Sequencing of *S10* region
Lm-S10-6	AATGAACCCGAACGATCACC	Sequencing of *S10* region
Lm-S10-7	TACAAGCGCAAAAGCCGTTG	Sequencing of *S10* region
Lm-S10-8	GTGCAGCTAACCGTGTGAAT	Sequencing of *S10* region
Lm-S10-9	AGGCGGAACTGAAGTTGCAT	Sequencing of *S10* region
Lm-spc-1	ACCCGTTATTCTGGAAGGAG	Amplification of *spc* region and sequencing
Lm-spc-R	AAGGCATTACACCCATGGCA	Amplification of *spc* region and sequencing
Lm-spc-F	CTCGTCCATTGTCTGCAACT	Sequencing of *spc* region
Lm-spc-2	CAAACGTAATGCTAMTTGACCC	Sequencing of *spc* region
Lm-spc-3	CGTGGTAACTATACGTTGGGT	Sequencing of *spc* region
Lm-spc-4	GACTGGCGAACGTGTAATCA	Sequencing of *spc* region
Lm-spc-5	TCCTGCAAACACWCAAGTGATT	Sequencing of *spc* region
Lm-spc-6	GGAGGGACATATTACATGCCTG	Sequencing of *spc* region
Lm-spc-7	TTAATCGGACGCCCTCAA	Sequencing of *spc* region
Lm-alpha-F	CTCTACCAAACGCGATGTTC	Amplification of *alpha* region and sequencing
Lm-alpha-R	GGAAACACAGAGCTAGACAAGG	Amplification of *alpha* region and sequencing
Lm-alpha-1	CCTGACACGCGGAAGAATTA	Sequencing of *alpha* region
Lm-alpha-2	AAGGCCCGTCCAAAACAGTA	Sequencing of *alpha* region
Lm-alpha-3	CAGCGATGATGCCAAGTATG	Sequencing of *alpha* region
Lm-alpha-4	GAAGCAGTTTCACTTGGAGC	Sequencing of *alpha* region
Lm-alpha-5	AACTGGCTGACCTTGGCTTA	Sequencing of *alpha* region
Lm-L21-F	CCCCTGTGATGGCGAGTCTT	Amplification of L21 and sequencing
Lm-L21-R	TCTTCTCGCATAACATCGACTTGAA	Amplification of L21
Lm-S21-F	TGAAGGATTTAAGTGAGTGCATGT	Amplification of S21 and sequencing
Lm-S21-R	CGCATCGCTTGTTTCATATCT	Amplification of S21
Lm-S9-F	TTCGGGAGCTAATTTGTTTCAA	Amplification and sequencing of S9 and L13
Lm-S9-R	AACGTTTTCAGAACTGAGGTGC	Amplification and sequencing of S9 and L13
Lm-S9-F2	CACATATCGACACTGGAGACTTTG	Sequencing of S9 and L13
Lm-L10-F	CTGGAATCAAAGTCGACCCA	Amplification of L10 and sequencing
Lm-L10-R	GCAGCAGTTACGCCAAATTCTT	Amplification of S21
Listeria_sp-L31-F	TGTTATAATATYTATACTGTGTGTAAAAGC	Amplification of L31 and sequencing
Listeria_sp-L31-R	TGAGACCGTAYTTTTTGTTGAAGC	Amplification of L31 and sequencing

### MALDI-TOF MS analysis

Bacterial cells grown overnight on an agar plate (three colonies) or in 2 mL liquid medium were collected and suspended in 0.5 mL of 70% (v/v) ethanol. Cells were then separated by centrifugation at 10,000 × *g* for 2 min at 4°C and dehydrated using a centrifugal evaporator (CVE-3100, EYELA, Tokyo, Japan), after which a 10 μL aliquot of 35% (v/v) formic acid and cells were mixed by pipetting. Then, 1.5 μL of this mixture was mixed with 10 μL matrix reagent containing 20 mg/mL sinapinic acid (SA; Wako Pure Chemical Industries, Osaka, Japan) and 1% trifluoroacetic acid (Wako Pure Chemical Industries) in 50% (v/v) acetonitrile; then 1.5 μL was spotted onto the analytical metal plate. Samples were analyzed using an AXIMA Microorganism Identification System (Shimadzu Corporation) with 100 laser shots at a spectrum range of 2000 *m*/*z*– 35000 *m*/*z* with 500 ppm tolerance. We used α-cyano-4-hydroxycinnamic acid (CHCA) as a matrix for the SARAMIS database searching, as described in a previous report [25]. For calibrating the instrument, *Escherichia coli* DH5α was used according to the manufacturer’s instructions.

### Analysis with Strain Solution software

The datasets of MS and peak intensity in ASCII files were incorporated into the software Strain Solution version 1.0.0. (Shimadzu Corporation) and analyzed according to the instructions. The MS values of the parental database were registered in the software in advance.

## Results

### Construction of an MS database for discriminating *L*. *monocytogenes* lineages

**Fig 1:**
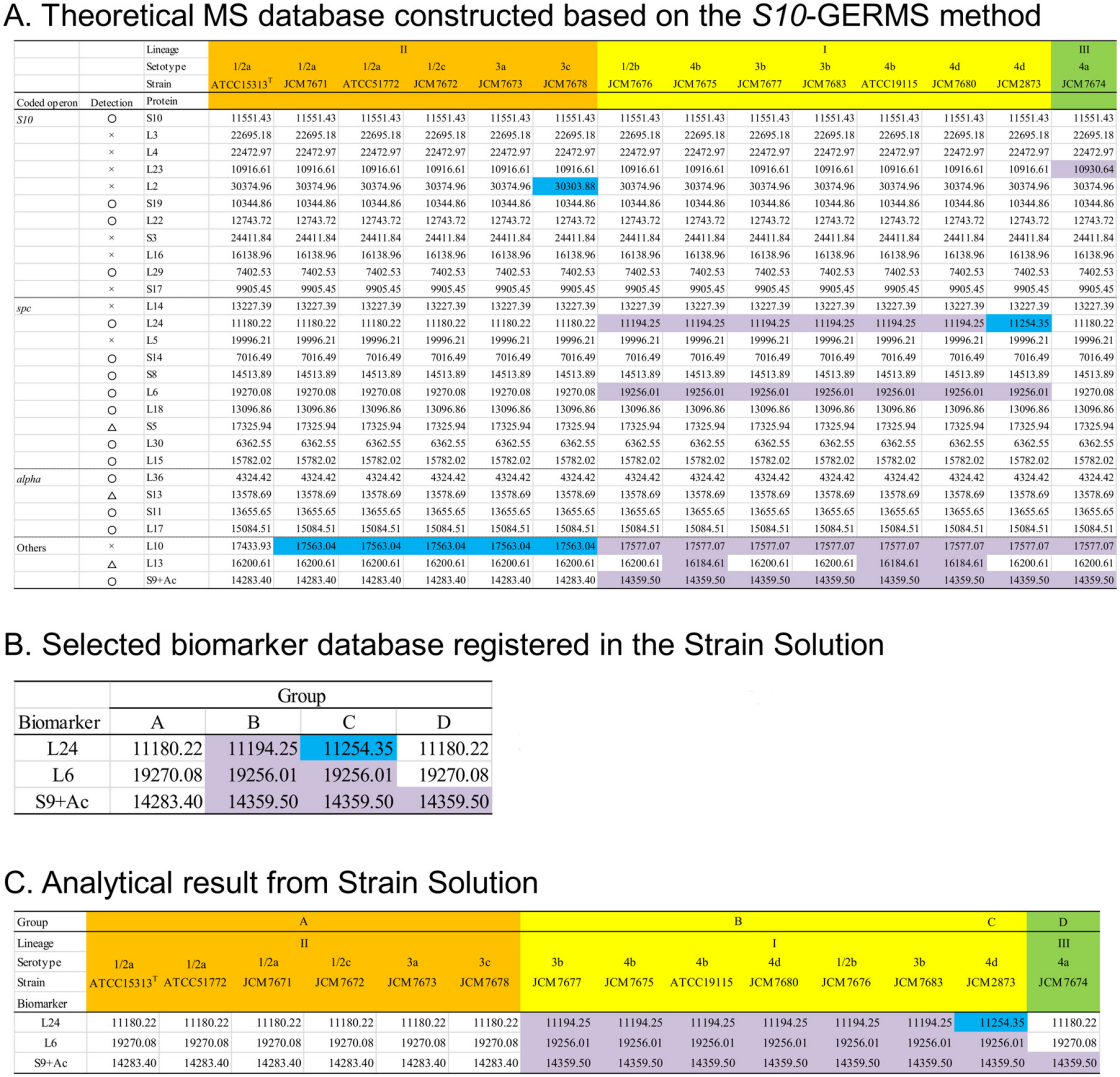
Theoretical MS database constructed with publically available *L*. *monocytogenes* strains and analysis with Strain Solution software. **A**. The theoretical *m*/*z* of the ribosomal proteins in *S10-spc-alpha* operon and an additional three biomarker candidates are shown. Circles in ‘Detection’ means the corresponding peaks are detectable in all strains with default analytical condition (threshold offset: 0.015 mV; threshold response: 1.200) in the AXIMA system. The undetected or weak peaks that we could not always find are marked with an “x”. Triangles indicate that MS differences in each strain or the other protein peaks could not be distinguished from one another with a 500 ppm tolerance, though putative peaks were detected. Characteristic MS values were colored. **B**. The selected three biomarkers registered as the standard database in Strain Solution. **C**. The results obtained from the Strain Solution analysis.

First, we constructed an *m*/*z* database of ribosomal proteins encoded in the *S10-spc-alpha* operon and additional potential biomarkers using publically available *L*. *monocytogenes* strains (Fig 1A). The ribosomal proteins L23, L2, L24, and L6 in the *S10-spc-alpha* operon and the three additional ribosomal proteins L10, L13, and S9 appear capable of differentiating serotypes of *L*. *monocytogenes* because their unique MS depending on the strains. From the comparison of the theoretically calculated MS of S9 and its actual observed MS peaks, the mass weight of the observed peak of S9 shifted +43, indicating an acetylated S9. Among these, L23, L2, and L10 were not detected in the MALDI-TOF MS analysis, although they were expected to be powerful biomarkers from their theoretical MS value varieties. We observed the MS peaks of L13 with *m*/*z* 16200.61 or 16184.61 in all *L*. *monocytogenes* samples and *m*/*z* 16187.61 in *L*. *seeligeri* strains (data not shown); however, this protein was not suitable as biomarkers because MS differences (shifts) were too small. In contrast, the three ribosomal proteins L24, L6, and S9 were ideal biomarker candidates because they were always detected, regardless of the strain, with the current MS detection tolerance (more than 500 ppm).

### Analysis of *L*. *monocytogenes* with Strain Solution software

From the results above, we selected three ribosomal proteins (L24, L6, and S9) as biomarker proteins capable of discriminating *L*. *monocytogenes* lineages. Four patterns of MS values of these proteins (listed in Fig 1B), which divided *L*. *monocytogenes* into four groups (A to D), were preregistered in the analytical software Strain Solution. First, 14 *L*. *monocytogenes* strains were identified as ‘*L*. *monocytogenes*’ using the conventional fingerprinting analysis software, SARAMIS (data not shown). Next, the MS data obtained with matrix SA was imported into the Strain Solution software. As shown in Fig 1C, the attribution of all biomarkers generated from automated analysis with Strain Solution were correct. The lineages of *L*. *monocytogenes* were classified into four groups (A to D) as follows: lineage I [registered group B (serotypes 1/2b, 3b, 4b, 4d, and 4e) and group C (serotype 4d)], lineage II [group A (serotypes 1/2a, 1/2c, 3a, and 3c)] and lineage III [group D (serotype 4a)]. Thus, we confirmed that these three biomarkers, L24 (*m*/*z* 11180.22, 11194.25, or 11254.35), L6 (*m*/*z* 19270.08 or 19256.01), and acetylated S9 (*m*/*z* 14283.40, 14359.50, or 14302.45), are useful to discriminate between lineages or serotypes of *L*. *monocytogenes* in MALDI-TOF MS using Strain Solution.

### Construction of an MS database for *Listeria* species

**Fig 2:**
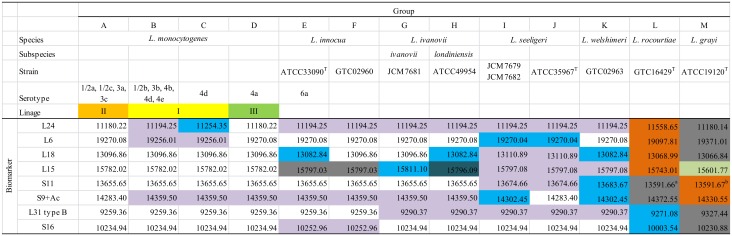
Theoretical MS database for *Listeria* species. ^a^The MS [M + H]^+^ plus *m*/*z* 17 of the theoretically calculated value is shown based on the observed MS peak. ^b^The MS [M + H]^+^ plus *m*/*z* 14 of the theoretically calculated value is shown because it appeared to be methylated.

As described above, three promising sets of ribosomal proteins (L24, L6, and S9) were consistently detected by MALDI-TOF MS analysis and were effective for typing of *L*. *monocytogenes*. From the analytical MS data obtained from MALDI-TOF MS, these biomarkers were detected in all *Listeria* spp. as well as *L*. *monocytogenes*(S1 Fig). Furthermore, we found that five additional ribosomal proteins, L18 (*m*/*z* 13096.86 or 13110.89), L15 (*m*/*z* 15782.02, 15797.08, or 15668.86), S11 (m/z 13655.65 or 13674.66), L31 type B (*m*/*z* 9259.36, 9290.34, 9327.44, or 9271.3), and S16 (*m*/*z* 10234.94, 10252.97, 10230.88, or 10003.54) gave characteristic MS patterns to the species of *Listeria*. S13 (*m*/*z* 13578.69 in *L*. *monocytogenes* or 13552.65 in *L*. *seeligeri*) was also detected, but one of the peaks overlapped with the peaks of L20 (*m*/*z* 13552.08) (data not shown). From these findings, we focused on eight biomarkers (L24, L6, L18, L15, S11, S9, L31 type B, and S16) and constructed a theoretical MS database for *Listeria* species using publically available strains (Fig 2). It should be noted that S11 in *L*. *rocourtiae* and *L*. *grayi* was post-translationally modified, while that of the others was not.

### Comparison of fingerprinting analysis and *S10*-GERMS method

As mentioned above, the conventional fingerprinting analysis software SARAMIS could properly identify all *L*. *monocytogenes* strains to the species level. Here, we analyzed *L*. *innocua*, *L*. *ivanovii*, *L*. *seeligeri*, *L*. *welshimeri*, *L*. *rocourtiae*, and *L*. *grayi* by SARAMIS. As shown in Table 4, most of them were identified as only ‘*Listeria* spp.’ except for *L*. *grayi*, which was correctly identified to the species level. However, *L*. *ivanovii* JCM7681, *L*. *seeligeri* JCM 7679 and JCM 7682 were misidentified as *L*. *monocytogenes*. While *L*. *rocourtiae* had a 0% match to the parental database in SARAMIS, resulting in ‘not identified’ at first, it was correctly identified to species level after an additional database was imported according to the manufacturer’s instructions for SuperSpectra^™^ in SARAMIS. This implies that fingerprinting depends greatly on the reference database quality. The parental database for *L*. *rocourtiae* registered here is shown in S1 Table.

**Table 4:** Results from fingerprinting analysis with SARAMIS.

Sample	Identity (%)	Family	Genus	Species
*L*. *innocua* ATCC33090^T^	91.8	Family IV *Listeriaceae*	*Listeria*	sp.
*L*. *innocua* GTC02960	99.9	Family IV *Listeriaceae*	*Listeria*	sp.
*L*. *ivanovii* GTC02961	84.4	Family IV *Listeriaceae*	*Listeria*	*monocytogenes*
*L*. *ivanovii* ATCC49954^T^	99.9	Family IV *Listeriaceae*	*Listeria*	sp.
*L*. *seeligeri* ATCC35967	91.8	Family IV *Listeriaceae*	*Listeria*	sp.
*L*. *welshimeri* GTC02963^T^	97.2	Family IV *Listeriaceae*	*Listeria*	sp.
*L*. *rocourtiae* GTC16429^T^	99.9^a^		*Listeria*	*rocourtiae*
*L*. *grayi* ATCC19120	99.9	Family IV *Listeriaceae*	*Listeria*	*grayi*
*L*. *seeligeri* JCM7679	81.1	Family IV *Listeriaceae*	*Listeria*	*monocytogenes*
*L*. *seeligeri* JCM7683	81.1	Family IV *Listeriaceae*	*Listeria*	*monocytogenes*

^a^The parental database for SuperSpectra^™^ in SARAMIS is shown in S1 Table.

Next we tried automated discrimination at the species level using eight biomarkers in the database of Fig 2. First, MS patterns belonging to the group A to K were preregistered in Strain Solution based on Fig 2. The *m*/*z* 15797.08, 15797.03, and 15796.09 of L15 were preset as *m*/*z* 15797.08 because the MS differences were too small to be detected on a normal MALDI-TOF MS system. Similarly *m*/*z* 19270.08 and 19270.04 (specific to *L*. *seeligeri*) of L6 were registered as *m*/*z* 19270.08 because their MS differences were too small to be recognized. As a result, all *Listeria* spp. strains listed in Table 1 were assigned to the proper groups by our system. The spectra of the biomarkers obtained in our MALDI-TOF MS analysis are shown in S1 Fig.

### Evaluation of the constructed MS database

Strains isolated from environment were used for blind test to evaluate the constructed MS database (Table 2). In fingerprinting analysis with SARAMIS, all *L*. *monocytogenes* strains (1–6 and 17) and *L*. *rocourtiae* (18–20) were correctly identified as *L*. *monocytogenes* and *L*. *rocourtiae* at the species-level, respectively. In contrast, the other 13 strains were just identified as *Listeria* spp., although the SARAMIS database has already included the *m*/*z* information of *L*. *innocua* and *L*. *ivanovii*.

The Strain Solution software further scanned *m*/*z* data of a total 20 strains, except for *L*. *rocourtiae*. The analytical results of the traditional method and Strain Solution are summarized in Table 5. Using eight biomarkers in Fig 2, all *L*. *monocytogenes* (seven strains), *L*. *innocua* (six strains), and *L*. *ivanovii* (three strains) were correctly identified at the lineage or species level in our system, with a 100% match for each group. However identification accuracy of *L*. *seeligeri* was 3/4 (75%) due to the misidentification of No. 13 strain into *L*. *innocua*.

**Table 5:** Identification results by traditional method and Strain Solution.

	Traditional method	Strain Solution	
Strain No.		Group	
1, 2, 3, 4, 17	*L*. *monocytogenes* serotype 4b (lineage I)	A	*L*. *monocytogenes* lineage I
5	*L*. *monocytogenes* serotype 1/2a (lineage I)	A	*L*. *monocytogenes* lineage I
6	*L*. *monocytogenes* serotype 1/2b (lineage I)	A	*L*. *monocytogenes* lineage I
7, 12	*L*. *innocua*	F	*L*. *innocua*
13	*L*. *seeligeri*	F	*L*. *innocua*
8, 9, 10, 11	*L*. *innocua*	E	*L*. *innocua*
14, 15, 16	*L*. *seeligeri*	I	*L*. *seeligeri*
21, 23	*L*. *ivanovii*	H	*L*. *ivanovii_*subsp. *londiniensis*
22	*L*. *ivanovii*	G	*L*. *ivanovii_*subsp. *ivanovii*

Strain No. and group are referred to Table 2 and Fig 2, respectively.

Traditional method is by 16S rRNA sequencing and antisera serotyping kit.

### Accession No

DNA sequences of genes encoding ribosomal proteins of *L*. *monocytogenes* in Fig 1, all of which were determined in this study for the first time, were registered in the DNA Data Bank of Japan (Mishima, Japan) as accession numbers LC104817 to LC104928. The sequences of *Listeria* spp. in Fig 2 were registered as accession numbers LC104929 to LC104975.

## Discussion

In this study, we constructed an accurate and reliable MS database to discriminate lineages of *L*. *monocytogenes* and species of *Listeria* in MALDI-TOF MS based on the proteotyping using a *S10*-GERMS method, which combines both genomics and proteomics. First, we constructed a database of ribosomal proteins encoded in the *S10-spc-alpha* operon for *L*. *monocytogenes* and found that the MS of 15 kinds of proteins varied with serotype (Fig 1). Among these, three potential biomarkers, L24, L6, and S9, whose MS peaks were always detected in MALDI-TOF MS analysis were selected as biomarkers for typing *L*. *monocytogenes*. In addition, five potential biomarkers, L18, L15, S11, L31 type B, and S16 with a specific MS value in *L*. *innocua*, could be used to differentiate *Listeria* species, including *L*. *monocytogenes* (Fig 2). CHCA is a recommended matrix reagent in fingerprinting methods; therefore, it is difficult to identify high molecular *m/z* proteins. However, when SA was used as a matrix reagent, the novel biomarkers with high *m/z* were successfully detected in this study as follows: ribosomal proteins L6 (*m*/*z* 19270.08 or 19256.01), L18 (*m*/*z* 13096.86 or 13110.89), L15 (*m*/*z* 15782.02, 15797.08, or 15668.86), S13 (*m*/*z* 13578.69 or 13552.65), S11 (*m*/*z* 13655.65 or 13674.66), acetylated S9 (*m*/*z* 14283.40, 14359.50, or 14302.45), L31 type B (*m*/*z* 9259.36, 9290.34, 9327.44, or 9271.3) and S16 (*m*/*z* 10234.94, 10252.97, 10230.88, or 10003.54). Moreover, previously reported MS peaks of *m/z* 11179, 11871.31, or 11199.33 by other groups [16, 19] were confirmed as solid values of peaks corresponding to the ribosomal protein L24 with *m*/*z* 11180.22, 11194.25, or 11254.35, respectively, by proteotyping for the first time. The differences in MS values between previous reports and this study come from the accuracy of the experimental procedure. To realize strain- or serotype-level microbial discrimination at a higher resolution than that of conventional fingerprinting analysis, the accuracy of MS values is important because it relies upon the MS database, which reflects even single amino acid substitutions. In fact, we often observe slight differences in MS peaks derived from the same proteins in closely related microorganisms [23, 25]. Fingerprinting analysis may still greatly be influenced by the culture and/or growing conditions of the target bacteria [30]; therefore, proteotyping data backed up by genetic sequences will be of great importance for correct identification. Our results indicate that ribosomal proteins L24, L6, and S9 are especially important to discriminate lineages of *L*. *monocytogenes* and to differentiate *L*. *monocytogenes* and *L*. *seeligeri* (Figs 1 and 2).

In contrast, the MS peaks of *m*/*z* 5590, 5594.85, 5601.21, and 6184.39, identified by previous reports as biomarkers for serotyping *L*. *monocytogenes* [16, 19], were observed in this study as *m*/*z* 5590 (lineages II and III) and *m*/*z* 5595 (lineage I), although serotype 4d strains did not exhibit the corresponding peaks (data not shown). We did not select this unknown protein as a potential biomarker because the peak intensities were insufficient in our analysis, likely due to the use of SA as the matrix.

*L*. *grayi* and *L*. *rocourtiae* were correctly identified at the species level by SARAMIS and SuperSpectra (Table 3). *L*. *grayi* is known to be evolutionarily distant from other *Listeria* spp. [31], and *L*. *rocourtiae* is a recently emerging species isolated from lettuce in Australia [32]. The MS values of the eight biomarkers in these two species differed greatly from those of the other *Listeria* spp. (Fig 2A). This result is consistent with the concept that ribosomal protein evolution is well associated with bacterial species [33]. However, SARAMIS could not identify them correctly at the species level due to their very similar MS profiles, which may be indistinguishable by conventional fingerprinting (Table 3). Even in such a case, the MS database in Fig 2 with the Strain Solution software plays a significant role in the success of discrimination.

We further analyzed environmental strains for blind test and validated the MS database (Tables 2 and 5). The hemolytic species *L*. *monocytogenes*, *L*. *seeligeri*, and *L*. *ivanovii* are genetically close [34, 35] and sometimes misidentified. In our study, one *L*. *seeligeri* strain (No. 13) was identified as *L*. *innocua* by Strain Solution. It was most likely to be *L*. *seeligeri* by 16S rRNA sequence analysis, but the profiling of physiological biochemical test was *L*. *innocua* or *L*. *welshimeri* due to the positive signal of arylamidase and d-xylose utilization (data not shown). These results supported by our discrimination result, suggesting that Strain Solution could distinguish such minor differences between very similar strains that might be misidentified or overlooked by traditional methods.

The main aim of this study was the construction of a standardized and reliable database for an important pathogen, the genus *Listeria*. We successfully demonstrated the capability of the constructed database and Strain Solution software to discriminate *L*. *monocytogenes* at serotype level, as well as different species of *Listeria* that were difficult to identify by conventional fingerprinting methods. Although we assessed this database using naturally isolated strains (Table 2), demonstration using larger scale samples still required for validation. Nevertheless, we believe that proteotyping software, Strain Solution, together with the accurate MS database constructed here can be broadly applied by any laboratory using any MALDI-TOF MS system to perform strain- or serotype-level microbial classification beyond conventional fingerprinting. Therefore, we are willing to evaluate the constructed database in collaboration with institutions possessing *Listeria* isolates. These investigations will open a new window to discriminate bacteria in clinical and diagnostic laboratories, and also food-related industries.

## Supporting Information

S1 Fig MS peaks of the selected eight biomarkers. Groups A to K correspond to that of Fig 2. Arabic numerals above the graph indicate the number of patterns.
(TIF)

S1 Table MS data of *L*. *rocouritiae* registered in SuperSpectra. The registration of entries refers to the manual of SuperSpectra.
(DOCX)
