# Supplementary figures and images for: Matrix-assisted Laser Desorption Ionization-Time of Flight Mass Spectrometry (MALDI-TOF MS) Can Precisely Discriminate the Lineages of Listeria monocytogenes and Species of Listeria
Source: PLoS One. 2016 Jul 21;11(7):e0159730. doi: 10.1371/journal.pone.0159730 (PMC4956195; doi:10.1371/journal.pone.0159730)

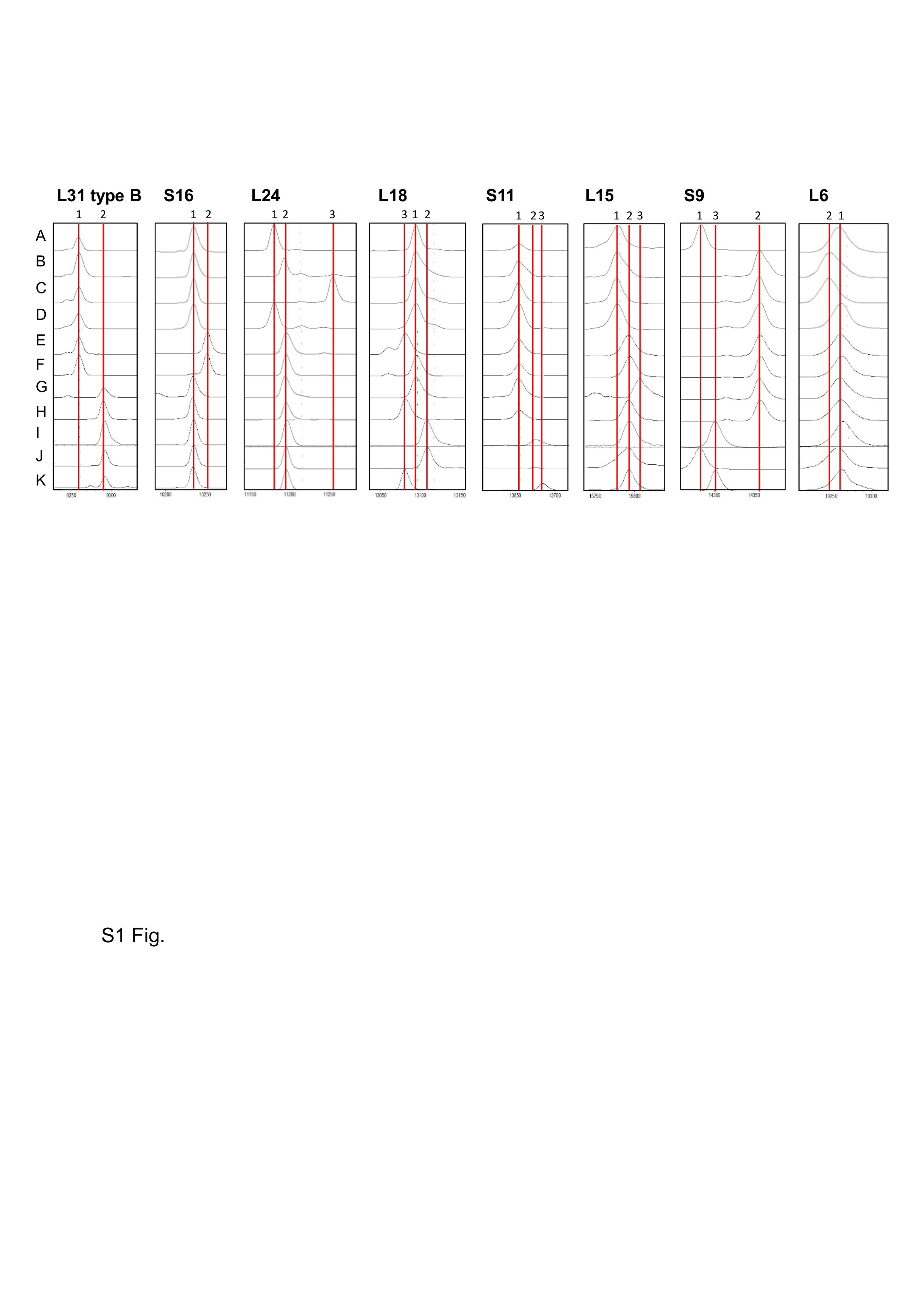

Supplement: S1 Fig — Groups A to K correspond to that of Fig 2. Arabic numerals above the graph indicate the number of patterns. (TIF) [file pone.0159730.s001.tif]
