# Supplementary material for: Matrix-assisted Laser Desorption Ionization-Time of Flight Mass Spectrometry (MALDI-TOF MS) Can Precisely Discriminate the Lineages of Listeria monocytogenes and Species of Listeria
Source: PLoS One. 2016 Jul 21;11(7):e0159730. doi: 10.1371/journal.pone.0159730 (PMC4956195; doi:10.1371/journal.pone.0159730)
Supplement: S1 Table — The registration of entries refers to the manual of SuperSpectra. (DOCX) [file pone.0159730.s002.docx]

S1 Table. MS data of *L. rocouritiae* registered in SuperSpectra.

| mass | error | rel. intensity | number_code |
| --- | --- | --- | --- |
| 3647.1 | 0.0173 | 16.9 | 30 |
| 3708.5 | 0.0134 | 8.0 | 30 |
| 4306.5 | 0.0076 | 1.2 | 30 |
| 4368.1 | 0.0070 | 2.5 | 30 |
| 4496.1 | 0.0091 | 4.8 | 30 |
| 4875.6 | 0.0075 | 34.9 | 100 |
| 5184.8 | 0.0052 | 8.1 | 30 |
| 5201.2 | 0.0073 | 7.7 | 30 |
| 6007.9 | 0.0002 | 40.6 | 100 |
| 6362.6 | 0.0021 | 70.7 | 100 |
| 6760.6 | 0.0009 | 3.8 | 100 |
| 6816.0 | 0.0087 | 4.1 | 30 |
| 7072.7 | 0.0027 | 19.5 | 30 |
| 7297.6 | 0.0018 | 71.1 | 30 |
| 7420.0 | 0.0021 | 25.5 | 100 |
| 8739.0 | 0.0025 | 12.0 | 30 |
| 8952.0 | 0.0005 | 2.7 | 30 |
| 8994.6 | 0.0005 | 22.6 | 30 |
| 9272.1 | 0.0011 | 24.9 | 100 |
| 9318.5 | 0.0000 | 16.9 | 30 |
| 9752.4 | 0.0027 | 99.1 | 100 |
| 9884.2 | 0.0011 | 28.4 | 30 |
| 10371.4 | 0.0004 | 15.3 | 100 |
| 10403.9 | 0.0006 | 12.1 | 100 |
| 12499.6 | 0.0064 | 3.4 | 30 |

The registration of entries is referred to the manual of SuperSpectra.
